# Biofertilizers mitigate salinity stress: insights from spring wheat physiology and gene expression

**DOI:** 10.3389/fpls.2025.1697160

**Published:** 2025-10-22

**Authors:** Halimah Al Khallaf, Hassan M. Rashad, Hameed Alsamadany

**Affiliations:** ^1^ Department of Biological Sciences, Faculty of Science, King Abdulaziz University, Jeddah, Saudi Arabia; ^2^ Department of Biological Sciences, College of Science, University of Jeddah, Jeddah, Saudi Arabia

**Keywords:** PGPR consortium, *Azotobacter chroococcum*, *Bacillus* spp., saline irrigation, antioxidant enzymes, stress-responsive gene expression

## Abstract

**Introduction:**

Salinity stress severely restricts plant growth and yield, reducing global crop productivity. Ensuring food security requires sustainable strategies to mitigate salinity damage. Beneficial microorganisms used as biofertilizers enhance plant tolerance to abiotic stresses. This study examined the response of spring wheat (*Triticum aestivum* L. cv. Yecora Rojo) to biofertilizers under varying salinity levels to assess their potential in enhancing salt stress tolerance.

**Methods:**

Three treatments were applied: untreated control (C), grain treatment (GT), and grain plus root treatment (GRT). Salinity stress was imposed using diluted seawater at 0, 2000, 4000, and 6000 ppm. The biofertilizer formulation included *Azotobacter chroococcum*, *Bacillus megaterium*, and *Bacillus circulans*. Physiological traits (chlorophyll, cell membrane stability, relative water content), biochemical markers (proline, malondialdehyde, hydrogen peroxide), and antioxidant enzyme activities (catalase, peroxidase, superoxide dismutase) were measured. Expression of salinity-responsive genes (TaCAT1, TaPOD-D1, TaSOD2, TaHKT1;4, TaNHX2, TaP5CS, TaFER-5B) was also analyzed.

**Results:**

Salinity significantly reduced wheat growth, chlorophyll levels, membrane stability, and water content. Biofertilizer treatments, especially GRT, alleviated these effects by maintaining chlorophyll and water status while reducing oxidative damage. Antioxidant enzyme activities increased, improving scavenging of reactive oxygen species. Biofertilizers also upregulated stress-related genes, enhancing osmotic adjustment, ion balance, and antioxidant defenses. Correlation analysis confirmed strong physiological and biochemical interactions supporting stress tolerance.

**Discussion & conclusion:**

Biofertilizers represent an eco-friendly and sustainable strategy to enhance wheat salinity tolerance. By boosting antioxidant defenses, osmolyte accumulation, and ion regulation, they mitigate salt-induced damage. GRT provided the greatest benefit, highlighting the synergistic effect of dual grain and root inoculation.

## 1 Introduction

One of the main abiotic stressors that significantly reduces wheat (*Triticum aestivum* L.) yield, particularly in arid and semi-arid conditions, is soil salinity. Reduced growth and yield are the results of high salinity’s detrimental effects on oxidative stress, ionic imbalance, and plant-water relations (Alotaibi et al., 2024). As a measure against such detrimental impacts, the utilization of rhizobacteria that are beneficial to the growth of plants (PGPR) as biofertilizers has become a promising, sustainable, and eco-friendly approach (Al Khallaf et al., 2024). PGPR induces salinity tolerance in plants through various mechanisms such as solubilization of nutrients, phytohormone production, and modulation of stress-associated pathways (Abu-Qaoud et al., 2021; Sadak and Dawood, 2023).

Recent research has accentuated the heightened efficacy of wheat co-inoculation with various strains of PGPR compared to single-strain inoculation (Tiwari et al., 2025). For instance, a consortium of *Ensifer adhaerens*, *Pseudomonas fluorescens*, and *Bacillus megaterium* greatly facilitated wheat growth attributes at salinity levels of up to 12 dS m^-1^ (Khan et al., 2022). In addition to increasing the K^+^/Na^+^ ratio and decreasing electrolyte leakage and sodium uptake, multi-strain inoculation led to improvements in shoot and root length, biomass production, chlorophyll content, and relative water content.

At the same time, co-inoculation of *Bacillus subtilis* and *Arthrobacter* sp. has been shown to ameliorate wheat salinity stress through the enhancement of dry biomass, soluble sugar, proline content, suppression of sodium accumulation and oxidative stress indicators. Such findings demonstrate the concerted activity of PGPR consortia in alleviating saline-induced damages (Otlewska et al., 2020).

*Azotobacter chroococcum*, a diazotrophic bacterium, promotes plant growth in saline conditions by increased nitrogen provision and secretion of growth-promoting factors. *Bacillus megaterium* and *Bacillus circulans*, with their phosphate-solubilizing abilities, increase phosphorus uptake in plants (Ayaz et al., 2022). Co-inoculation of these PGPR strains has the potential to improve nutrient acquisition, hormonal balance, and stress tolerance of wheat under saline conditions (Chaudhary et al., 2013).

The present study hypothesizes that the combination of *Azotobacter chroococcum*, *Bacillus megaterium*, and *Bacillus circulans* will result in a significant improvement in the resistance of spring wheat to salinity stress caused by seawater irrigation. The hypothesis will be achieved by enhancing the plant’s physiological resilience, growth performance, and overall vigor.

## 2 Materials and methods

In the current study, spring wheat (*Yecora Rojo*) was used, which is known for its moderate tolerance to salinity. The grain was obtained from the National Organization for Agricultural Services and Seed Production (BUTHOR) in Saudi Arabia. The study aimed to evaluate the effects of biofertilizer treatments and salinity concentration on the number of dependent variables in the wheat cultivar used. They were measured across four levels of salinity concentration, 0, 2000, 4000, and 6000 ppm, and three levels of biofertilizer treatments: Control (C), Grain Treatment (GT), and Grain and Root Treatment (GRTs). The experimental setup consisted of a randomized factorial design, and there were three separate replications. (4×3×3). The first factor is salinity concentration, which was applied through four dilutions of 0, 2000, 4000, and 6000 ppm of seawater. The second factor was co-inoculation with BF (*Azotobacter chroococcum*, *Bacillus megaterium*, and *Bacillus circulans*) at three levels (C (0g), GT (1g), and GRTs: (1g+5ml)). The study, therefore, included 12 treatments.

**Figure 1:**
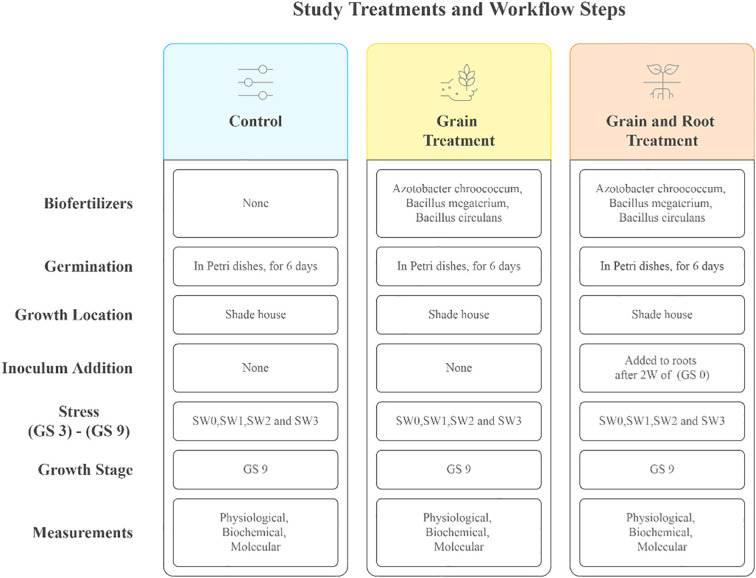
Experimental treatments and stages of implementation: Overview of biofertilizer applications and salinity stress levels the treatments include four salinity concentrations applied via seawater (SW0: 0 ppm, SW1: 2000 ppm, SW2: 4000 ppm, SW3: 6000 ppm) and three biofertilizer strategies (C, Control; GT, Grain Treatment; GRTs, Grain and Root Treatment). Growth stages of wheat (GS 0: germination, GS 3: third leaf, GS 9: flowering).

The experiment began with germination. It consisted of placing treated and control grains in petri dishes (10 grains in each) and adding 10 ml of distilled water to each dish, with a further 3 ml added later if necessary. After six days in a germination chamber, germinated grains were transferred to pots (plastic pots each measuring 26.5 cm in diameter and 22.5 cm in height) containing sterilized soil. Soil was prepared by mixing sand and peat moss in a 1:1 ratio, and it was sterilized using LabTech LAC-S autoclave at 121°C- 1 hour by placing a known weight in autoclave sterilization bags (Mahmood et al., 2014). Salinity stress began to be applied at the third leaf stage (GS 3) and continued until the flowering stage (GS 9), at a rate of twice a week with seawater dilutions and irrigation with plain water for the control group. The study was conducted in a shade house equipped with a polycarbonate roof and exposed to the natural conditions of Jeddah in the university area, from January to March 2024 (**Figure 1**).

### 2.1 Biofertilizers composition and application

The biofertilizers used in this study-*Azotobacter chroococcum* (N-fixing), *Bacillus megaterium* (P-solubilizing), and *Bacillus circulans* (K-solubilizing) were obtained from the Agricultural Research Center, Egypt. *A*. *chroococcum* was cultured on nitrogen-free media (Ashby’s Mannitol Agar) and formulated with a carrier for stability (Vessey, 2003). *B*. *megaterium* was isolated using Pikovskaya’s agar, mass-cultured in Pikovskaya’s broth, and combined with a sterile carrier to enhance phosphorus availability (Serag et al., 2019). *B*. *circulans* were sourced from plant rhizospheres, cultivated in nutrient-rich media, and formulated with carrier materials to promote plant growth through hormone production and nutrient solubilization (Meena et al., 2016). To prepare the BF treatment for co-inoculation, it was adding 1 g of each type was added, giving a total of 3 g, and this mixture was applied to the grains (Bhattacharyya and Jha, 2012). In addition, a bacterial suspension was prepared by adding 1 g of each type of biofertilizer (BF) to 1000 ml of sterile distilled water. This suspension was then applied directly to the root zone of the growing plants according to Vessey (2003) and Bashan et al. (2004).

### 2.2 Inoculation of grains and roots

Grain inoculation: All equipment was sterilized under UV light in a laminar flow hood cabinet prior to use. Wheat grains were surface sterilized by sequential treatment with 95% ethanol (2 min), followed by a 5% sodium hypochlorite (1 min), and then thoroughly washed with sterilized distilled water (Azmat et al., 2020). To apply the inoculant, the grains were first immersed in 2–5 mL of 15% sucrose solution Bashan (1998), and then treated with 1 g of biofertilizer (BF) containing 10^8^-10^9^ (Colony forming units; CFU) g^-1^ (Bashan et al., 2014).Then, the steps of grain inoculation were applied according to (Amjad et al., 2015).

Root inoculation of the growing plants was initiated by preparing a biofertilizer suspension by adding 1 g each of *A. chroococcum*, *B. megaterium*, and *B. circulans* to 1 L of sterile distilled water, following recommended dilution procedures to facilitate injection (Bhattacharyya and Jha, 2012). The resulting bacterial suspension contained approximately 10^8^−10^9^ CFU mL^-1^. After two weeks of germination (GS 0), an inoculum suspension of 5 mL per young plant was applied to the root zone using a root injector (Kumar et al., 2017). To ensure even distribution, the suspension was injected in five zones around the rhizosphere. A light irrigation was then applied to promote microbial survival and establishment in the rhizosphere and to aid in the microbes’ dispersion throughout the root zone (Bashan et al., 2004).

### 2.3 Dilutions and measurements of seawater

Diluted seawater solutions were prepared in concentrations of 0, 2000, 4000, and 6000 ppm, using seawater sourced from the Red Sea. The dilutions were prepared using Olsen (1986) formula. The hydrogen potential (pH) by a Man-Tech PC-1300–475 E pH meter, electrical conductivity (EC) ppm by a Hanna HI 5521 EC meter, cations and anions (mgL^-1^) by (ICP-OES, Varian 720-ES) in both tap water and seawater were analyzed.

### 2.4 Data collection

#### 2.4.1 Physiological traits

Using the Sattar et al. (2020) approach, the chlorophyll content (Chl) of the leaves was ascertained. Fresh leaf samples (0.5 g) were first weighed, homogenized in 80% acetone to remove the chlorophyll pigments, and then centrifuged for 10 minutes. A spectrophotometer was used to measure absorbance at 663 nm for chlorophyll (a) and 645 nm for chlorophyll (b) in the collected supernatant. The total amount of chlorophyll was then calculated. Chlorophyll concentrations were determined using Arnon’s formulae and absorbance values (1949).


Chlorophyll a=[12.7(A663)–2.69(A645)]

Chlorophyll b=[22.9(A645)–4.68(A663)]

Total Chlorophyll=[20.2(A645)+8.02(A663)]

The technique outlined by ElBasyoni et al. (2017) was employed to assess cell membrane stability (CMS). To determine the relative water content (RWC) according to Shah et al. (2022) fresh leaves were collected, and their fresh weight (FW) was recorded. Then the leaves were allowed to stay in distilled water for 4 to 6 hours, until they reached full turgidity. After this time, blotted the leaves gently to remove surface moisture and measured the turgid weight (TW). Then, the leaves were dried in an oven at 70 °C for 24 hours to obtain a constant dry weight (DW). Calculate the RWC using the formula:


RWC(%)=[(FW–DW)/(TW–DW)×100].

#### 2.4.2 Biochemical traits

For proline content (Pro) in plant tissues, a method was used Ábrahám et al. (2010) involves proline extraction from fresh leaf tissue using 3% sulfosalicylic acid. The extract is then reacted with an acid ninhydrin solution, which consists of ninhydrin dissolved in a mixture of glacial acetic acid and 6 M phosphoric acid. This reaction mixture is heated to facilitate the formation of a chromophore, which is then extracted using toluene. The toluene layer is measured spectrophotometrically with a UV–Vis spectrophotometer at 520 nm for absorbance to determine proline concentration.

Glycine betaine (GB) content was quantified by homogenizing fresh leaf tissue in distilled water, followed by centrifugation to obtain a clear supernatant. The extract was reacted with a potassium iodide-iodine reagent at room temperature, and the resulting complex was extracted using (1,2-dichloroethane). After phase separation by centrifugation, the organic phase was collected, and absorbance was measured at 365 nm using a spectrophotometer. The GB concentration was determined from a standard curve of known glycine betaine concentrations (Valadez-Bustos et al., 2016).

The activities of three key antioxidant enzymes—catalase (CAT), peroxidase (POD), and superoxide dismutase (SOD)—were evaluated following the protocol outlined by Djanaguiraman et al. (2020). For this, 2 grams of consistently frozen leaf tissue were homogenized with 0.1 M Tris-HCl buffer. The homogenate was then centrifuged at 2,000 rpm for 15 minutes at 4 °C. Enzymatic activities in the resulting supernatant were quantified using specific commercial assay kits: CAT activity was measured at 240 nm (Sigma-Aldrich, USA), POD at 470 nm (BiolabsInc, USA), and SOD at 560 nm (Sigma-Aldrich, USA).

Hydrogen peroxide (H_2_O_2_) content was determined according to the method described by Velikova et al. (2000). Lipid peroxidation levels were evaluated by measuring malondialdehyde (MDA) concentration using the thiobarbituric acid reactive substances (TBARS) assay. For this, 0.5 mL of fresh tissue extract was combined with 3 mL of 0.67% TBA, 1 mL of 20% TCA, and 0.5 mL of 0.25 N HCl. The mixture was boiled for 10 minutes, allowed to cool, and centrifuged. Absorbance was recorded at 532 nm, and MDA concentration was calculated using an extinction coefficient of 1.56 × 10^5^ M^-1^ cm^-1^, providing a reliable indicator of lipid oxidative damage (Reilly and Aust, 1999).

#### 2.4.3 Molecular traits

Total RNA was extracted from the selected wheat leaf samples using an RNA extraction kit from Sigma Aldrich. This was done in accordance with the standard protocol by Li et al. (2019) for the expression analysis of salinity-responsive genes (*TaSOD2*, *TaPOD-D1*, *TaCAT1*, *TaHKT1;4, TaNHX2, TaP5CS*, and *TaFER-5B*). Following this, cDNA synthesis was performed according to Ahmed et al. (2022). In this case, qRT-PCR analysis was performed with 2µg of total RNA. The expression levels of the genes of interest were calculated in relation to the reference gene TaActin1. **Table 1** contains the primer sequences used in the analysis.

**Table 1:** Primers used in the estimation of relative gene expression.

Gene	Primers
*TaSOD2*	CGCAGGACAACCAATGGACC (F) CGGAGGCACACTAGGCATCC (R)
*TaPOD-D1*	AGCACACAAGGAGAGAGGAG (F) AAGAGGCACGCGGTAGTCG (R)
*TaCAT1*	GGCCGCGCCGGAAACTGC (F) CGGGAACGAGAGGGCGAGAAAGA (R)
*TaHKT1;4*	AGCAAGCTGAAGTTGAGGGG (F) AGAGTTGTGACAGAGCCGTG (R)
*TaNHX2*	CTCAAGGGTGACTACCAAGCA (F) CCAATGCATCCATCCCGAC (R)
*TaP5CS*	GAAGGCTCTTATGGGTGTACTCAA (F) TAAAAGACCTTCAACACCCACAGG (R)
*TaFER-5B*	GCGTGGACCGTTGCTGCAACT (F) GGGCATCGCCTTTCTCAGCA (R)
*TaACTIN*	TACTCCCTCACAACAACC (F) GCTCCTGCTCATAATCAAG (R)

F, forward; R, reverse.

### 2.5 Analysis of statistics

Data collection and analysis were conducted using statistical software tools. All experiments were performed in three replicates. Analysis of variance (ANOVA) was carried out with Statistix 8.1, setting the significance level at p ≤ 0.05. Principal component analysis (PCA), correlation matrices, and heatmap visualizations were created using R software (version 4.1.0; The R Foundation for Statistical Computing, Vienna, Austria), following the methods described by Analytical Software (2008) and R Core Team (2021).

## 3 Results

### 3.1 Chemical composition of seawater

The analysis in **Table 2** shows stark differences between tap water and seawater. Tap water has low salinity (EC 75 ppm) with minimal ions, mainly Na^+^ (10 mgL^-1^) and Ca²^+^ (11 mgL^-1^), and low chloride (15 mgL^-1^) and sulfate (8 mgL^-1^). In contrast, seawater has extremely high salinity (EC 37,781 ppm), with abundant Na^+^ (12,166 mgL^-1^), Mg²^+^ (1,610 mgL^-1^), Cl^-^ (22,300 mgL^-1^), and SO_4_^2-^ (3,686 mgL^-1^). Despite both having a neutral pH (7.8), seawater’s high ionic content gives it a highly saline nature.

**Table 2:** Chemical analysis of tap water and seawater.

Source	pH	EC ppm	Cations (mg L^-1^)				Anions (mg L^-1^)	
			K^+^	Na^+^	Ca^2+^	Mg^2+^	Cl^-^	SO_4_^2-^
Tap water	7.8	75	0	10	11	0	15	8
Seawater	7.8	37781	502	12166	530	1610	22300	3686

### 3.2 Physiological traits

**Figure 2:**
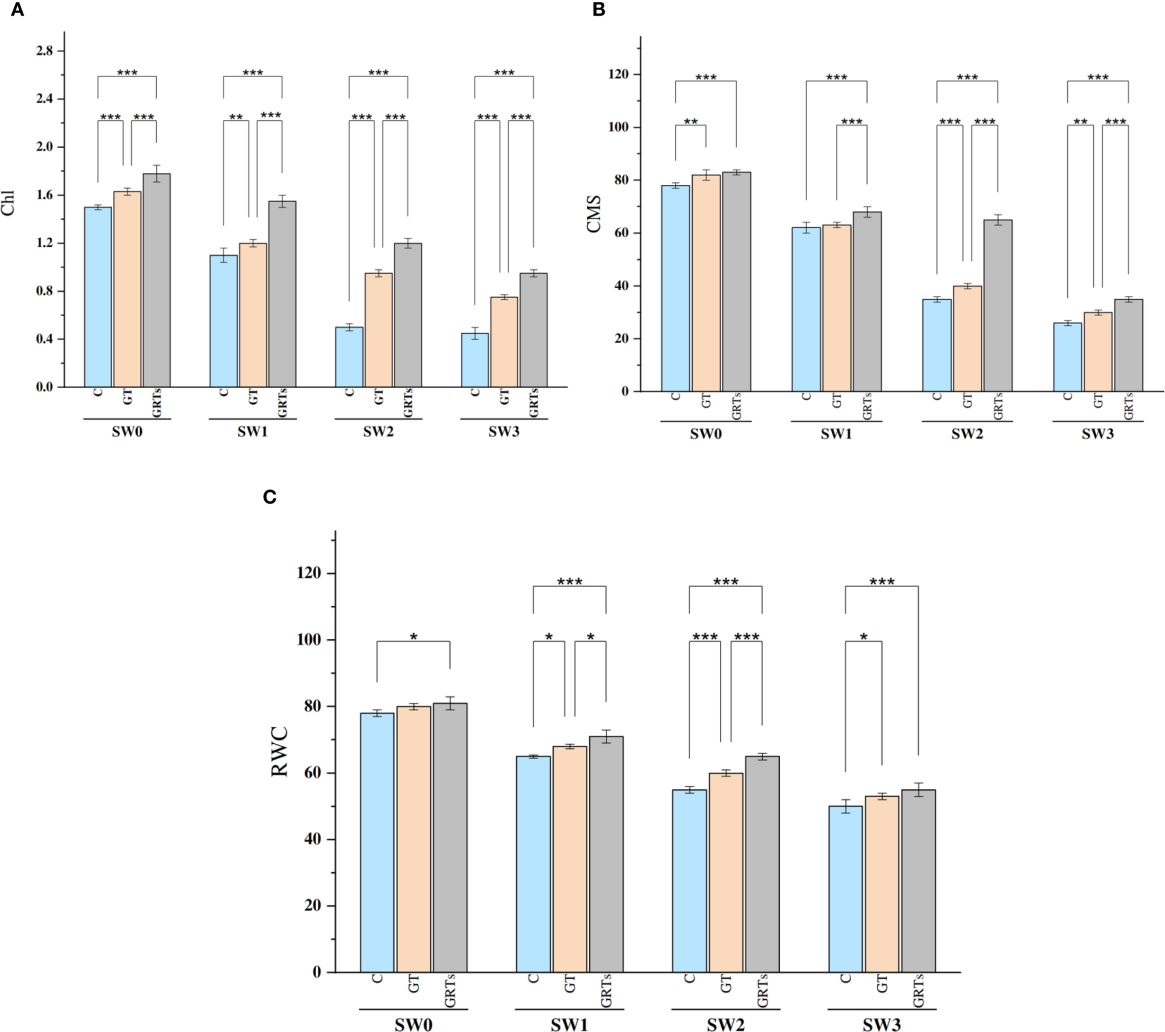
Effect of different concentrations for salinity stress by seawater (SW) 0,2000,4000 and 6000 ppm with biofertilizers (BF) treatments (C, control; GT, grain treatment; GRTs, grain and root treatment) on content of total chlorophylls (Chl) *g Kg^-1^*, cell membrane stability (CMS) *%* and relative water content (RWC)% in used wheat cultivar (*Yecora rojo*) at flowering stage **(A-C)**. Values in the graph are means averaged after 6 weeks of stress application at a p-value ≤ 0.05. Statistical significance is indicated by asterisks (*p ≤ 0.05, **p ≤ 0.01, *p ≤ 0.001).

All biofertilizer treatments had a statistically significant effect (p ≤ 0.05) on physiological parameters such as total chlorophyll content (Chl), cell membrane stability (CMS), and relative water content (RWC) in the examined wheat cultivar under different salinity conditions compared to the untreated control. Both Chl and CMS showed significant variations in response to salinity levels. An increase in salinity led to a marked reduction in Chl and CMS, with the highest values observed at SW3 and the lowest at SW0 (**Figures 2A, B**). Similarly, RWC declined significantly as salinity stress intensified, reaching its peak at SW3 (**Figure 2C**).

Conversely, Chl, CMS, and RWC values significantly improved with increasing levels of biofertilizer application from the control (C) to the GRTs treatment, while a slight reduction was observed under GT compared to GRTs. Notably, under the interaction between salinity and biofertilizer (salinity × BF), the GRTs treatment consistently enhanced Chl, CMS, and RWC across all salinity levels. Overall, despite the improving effects of biofertilizers, increasing seawater concentration led to a progressive decline in Chl, CMS, and RWC in wheat.

### 3.3 Biochemical traits

**Figure 3:**
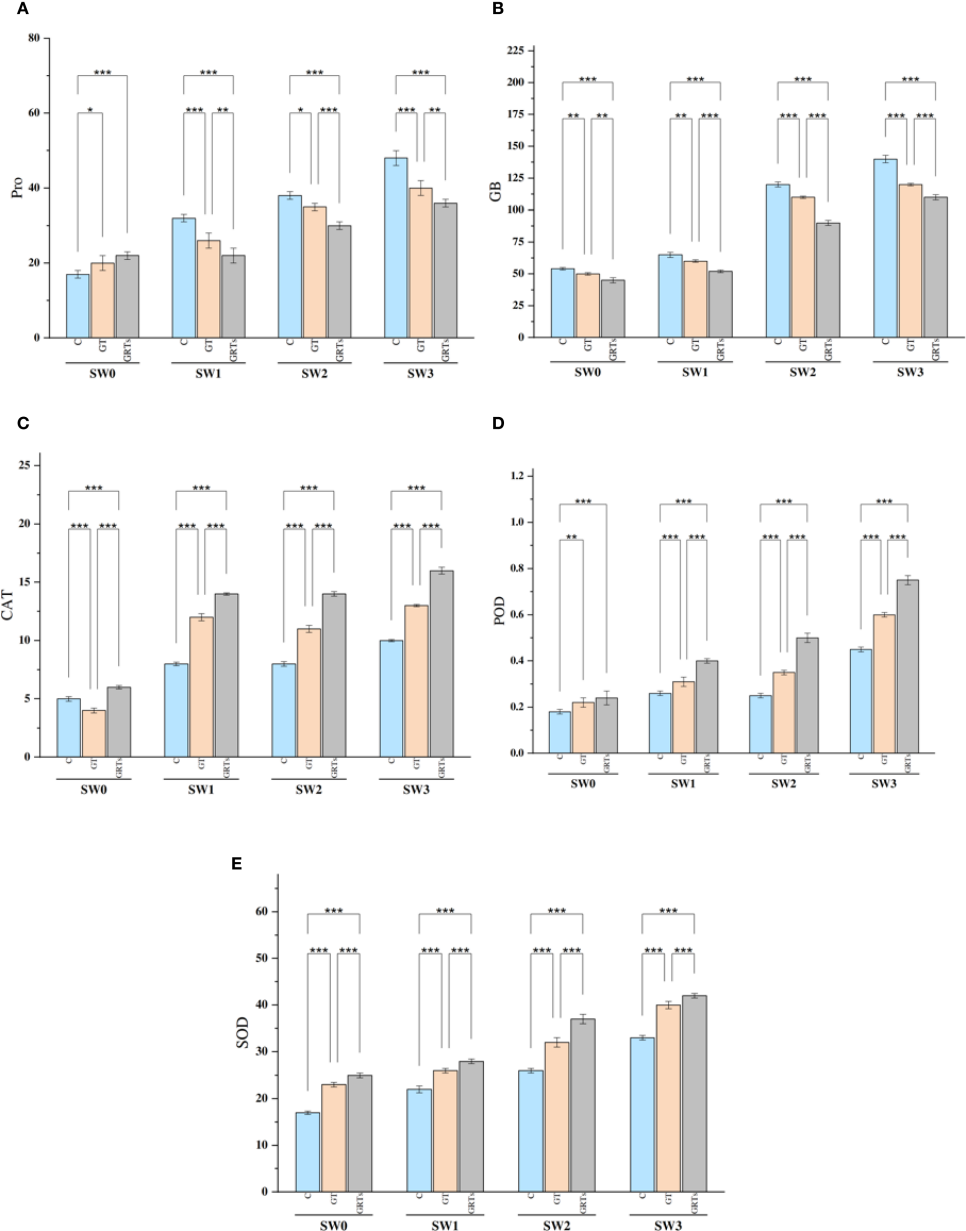
Effect of different concentrations for salinity stress by seawater (SW) 0,2000,4000 and 6000 ppm with biofertilizers (BF) treatments (C, control; GT, grain treatment; GRTs, grain and root treatment) on content of proline (Pro) *μgg^-1^ FW*, glycine betaine (GB) *μgg^-1^FW*, and antioxidant enzymes activity of catalase (CAT), peroxidase (POD) and superoxide dismutase (SOD) *Enzyme units* in used wheat cultivar (*Yecora rojo*) at flowering stage **(A–E)**. Values in the graph are means averaged after 6 weeks of stress application at a p-value ≤ 0.05. Statistical significance is indicated by asterisks (*p ≤ 0.05, **p ≤ 0.01, *p ≤ 0.001).

The buildup of osmolytes like proline (Pro) and glycine betaine (GB) was notably affected by the combined and separate impacts of salinity and biofertilizer (BF) treatments. An observable rise in Pro and GB content detected alongside increasing salinity levels, peaking at SW3 and hitting their lowest points at SW0. On the other hand, raising the level of BF treatment from C to GRTs led to a notable decrease in these osmolytes. Additionally, the combined effects of salinity and BF (salinity × BF) led to a significant reduction in Pro and GB concentrations under the GRTs treatment across various salinity levels. Generally, the levels of Pro and GB increased progressively with rising salinity in the wheat cultivar examined (**Figures 3A, B**), with the lowest concentrations observed under the GRTs treatment.

The activity of antioxidant enzymes—CAT, POD, and SOD—showed notable variation as a result of both independent and combined influences from salinity and BF treatments. There was a notable increase in enzyme activity as salinity levels rose. There was a steady increase in antioxidant enzyme activity observed from C to GRTs, with a significant rise noted at the GRTs treatment level (**Figures 3C–E**). Furthermore, the interaction between salinity and BF treatments resulted in significant changes in enzyme kinetics, with GRTs treatments exhibiting enhanced activity of CAT, POD, and SOD at all four salinity levels. Overall, in all BF treatments (C, GT, and GRTs), the activities of antioxidant enzymes rose as seawater concentrations increased in wheat.

**Figure 4:**
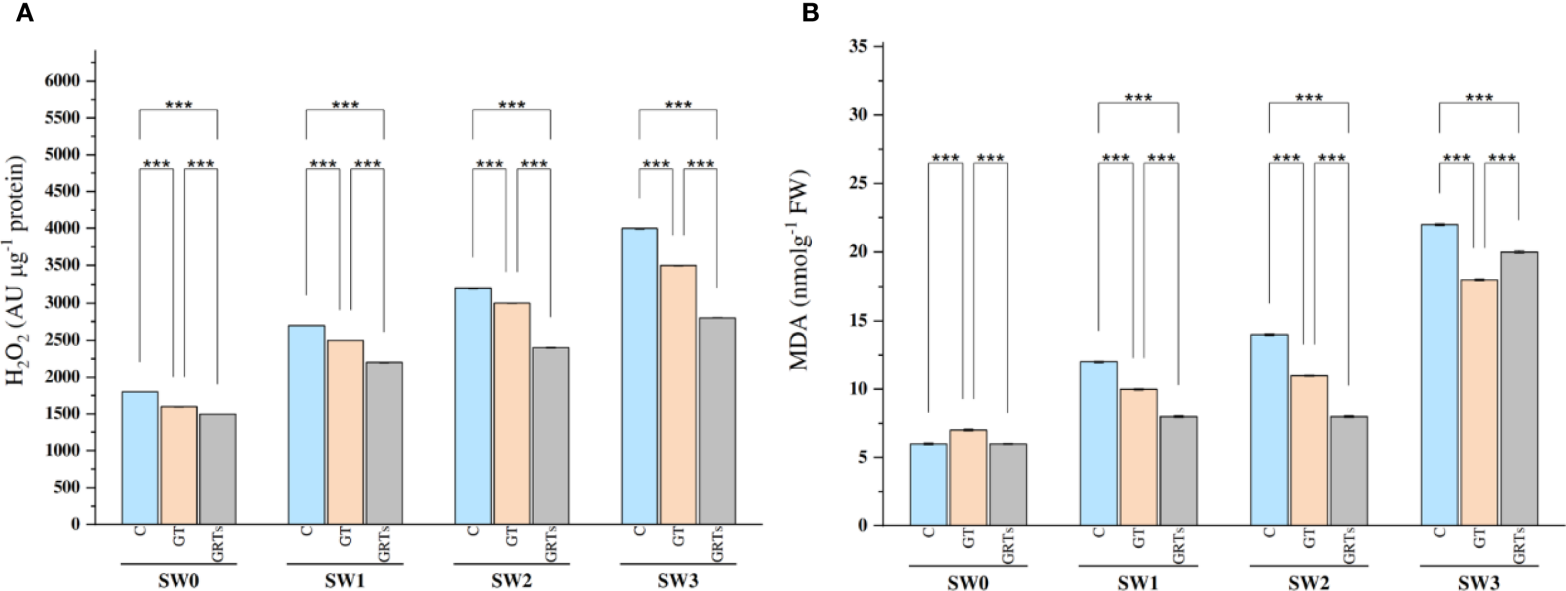
Effect of different concentrations for salinity stress by seawater (SW) 0,2000,4000 and 6000 ppm with biofertilizers (BF) treatments (C, control; GT, grain treatment; GRTs, grain and root treatment) on content of Hydrogen peroxide (H_2_O_2_) *AUμg^-1^ protein* and malondialdehyde (MDA) *nmolg^-1^FW* in used wheat cultivar (*Yecora rojo*) at flowering stage **(A, B)**. Values in the graph are means averaged after 6 weeks of stress application at a p-value ≤ 0.05. Statistical significance is indicated by asterisks (*p ≤ 0.05, **p ≤ 0.01, *p ≤ 0.001).

The levels of reactive oxygen species (ROS), especially hydrogen peroxide (H_2_O_2_), Responded markedly to both BF and salinity treatments. Salinity stress led to a significant increase in H_2_O_2_ levels, with the highest concentration recorded at SW3 (6000 ppm) and the lowest at SW0 (**Figure 4A**). However, the application of BF treatments contributed to a marked reduction in H_2_O_2_ levels. Under the combined effect of salinity and BF, the GRTs treatment significantly decreased H_2_O_2_ concentrations across all salinity levels. Overall, H_2_O_2_ levels increased proportionally with salinity in all BF treatments. Similarly, salinity stress significantly increased MDA levels, with the highest content recorded at SW3 and the lowest at SW0. The application of BF treatments, especially GRTs, markedly reduced MDA accumulation under all salinity conditions (**Figure 4B**).

### 3.4 Molecular traits

**Figure 5:**
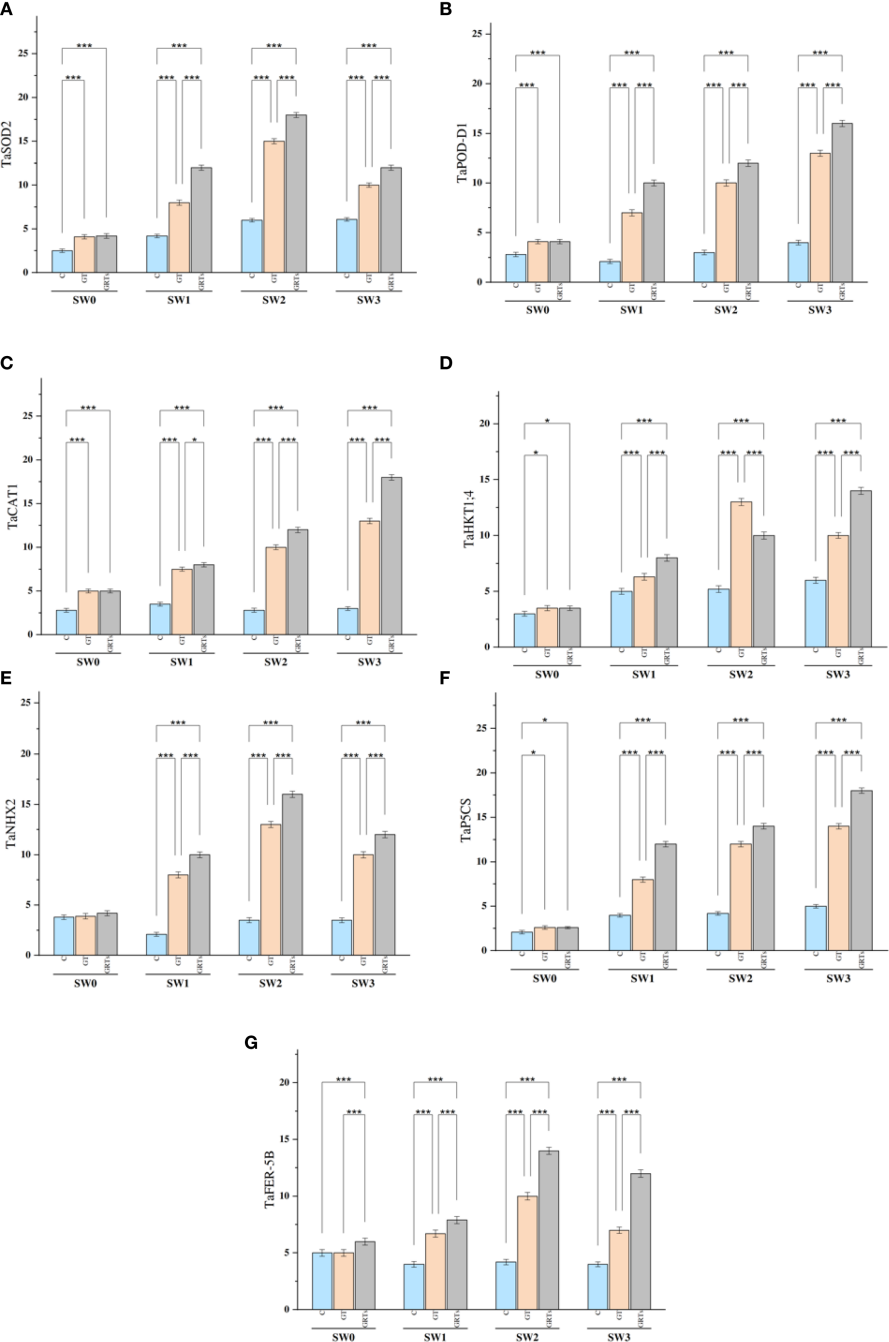
Varying expression patterns of salinity associated genes (*TaSOD2*, *TaPOD-D1*, *TaCAT1*, *TaHKT1;4*, *TaNHX2*, *TaP5CS and TaFER-5B*) under the control treatment, grain treatment, and grain and root treatment by applied biofertilizers under diluted seawater salinity stress in used wheat cultivar (*Yecora rojo*) at flowering stage **(A–G)**. Values in the graph are means averaged after 6 weeks of stress application at a p-value ≤ 0.05. Statistical significance is indicated by asterisks (*p ≤ 0.05, **p ≤ 0.01, *p ≤ 0.001).

The relative expression of the TaSOD2 gene Displayed notable statistical differences (p ≤ 0.05) under saline conditions as a result of varying biofertilizer (BF) treatments (**Figure 5A**), compared to the control. Across all levels of salinity, the GRTs treatment consistently produced the highest transcript abundance of TaSOD2, whereas the control (C) yielded the lowest. This expression pattern closely aligned with the activity of the SOD enzyme.

The expression profiles of the *TaPOD-D1* and *TaCAT1* genes revealed significant heterogeneity (p ≤ 0.05) in response to varying salinity levels and BF treatments (**Figures 5B, C**). Both genes exhibited peak expression with GRTs treatment at each salinity level, whereas the lowest expression levels were observed under the control, followed by GT. The gene expression patterns corresponded with the enzyme activities of POD and CAT, respectively. The overexpression of *TaSOD2*, *TaPOD*-*D1*, and *TaCAT1* appears Fundamental in reducing stress impact by modulating the levels of malondialdehyde (MDA) and reactive oxygen species (ROS).

Regarding ion transport-related genes, TaHKT1;4 and TaNHX2, which are key regulators of potassium influx, showed significant transcript variation (p ≤ 0.05) in response to the interactive effects of salinity and BF treatments (**Figures 5D, E**). Expression of both genes was highest under the GRTs treatment across most salinity levels, followed by GT and C, except in the case of *TaHKT1*;4 under SW2, where GT showed peak expression. These findings agreed with the measured K^+^ influx at corresponding BF and salinity levels.

The TaP5CS gene, responsible for proline biosynthesis, likewise exhibited notable expression changes (p ≤ 0.05) across all salinity treatments due to BF application (**Figure 5F**). GRTs yielded the maximum observed levels of *TaP5CS* expression, followed by GT and C, mirroring the trend observed in proline accumulation under identical treatment conditions.

In the case of ROS detoxification, TaFER-5B exhibited its highest expression under GRTs across all salinity levels, except at SW1, where GT demonstrated comparatively increased upregulation (**Figure 5G**). The expression pattern of TaFER-5B closely corresponded with reduced H_2_O_2_ content, suggesting its vital role in enhancing oxidative stress tolerance under saline stress conditions.

### 3.5 Analysis of correlation, principal component analysis, and heatmap

**Figure 6:**
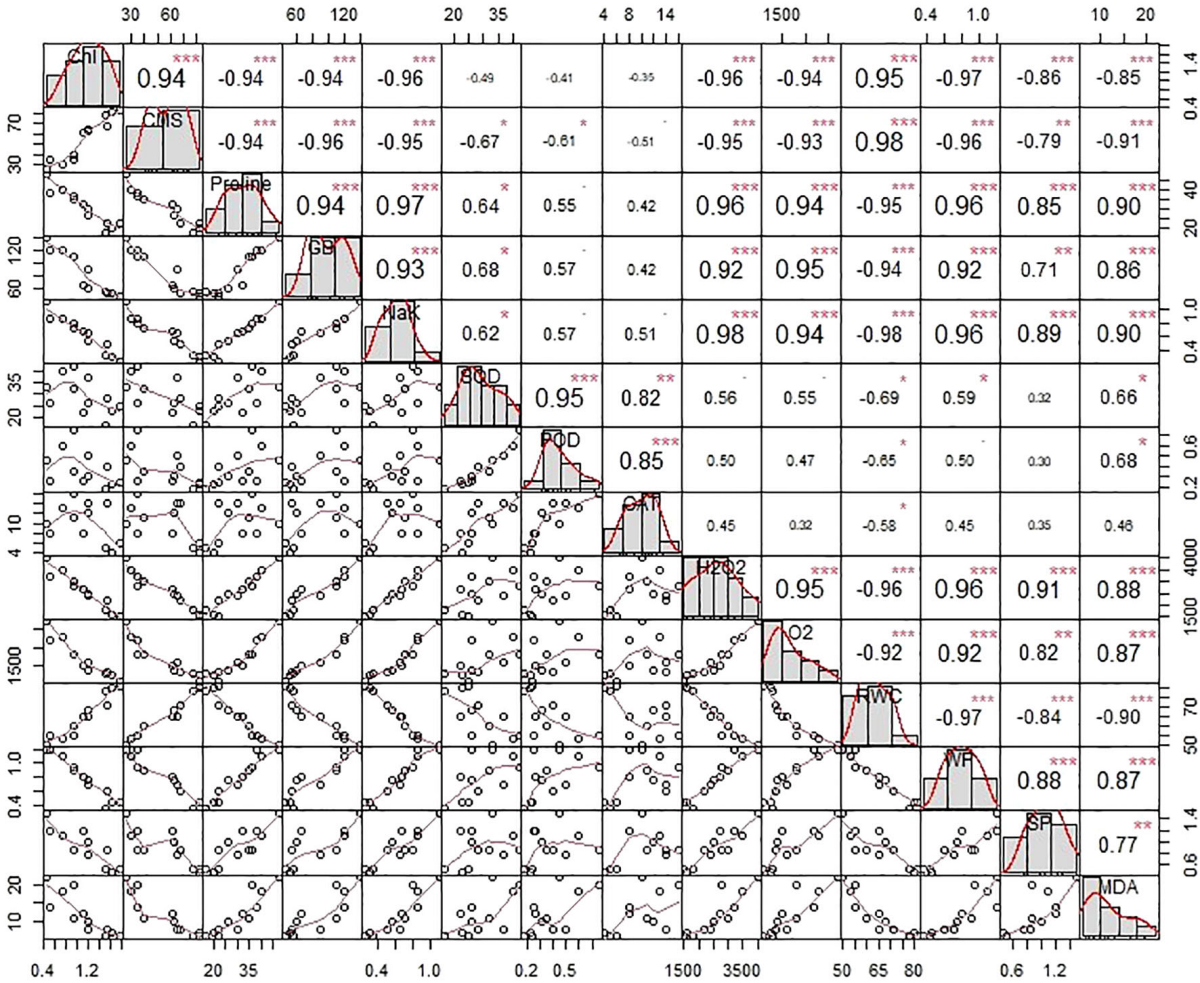
The correlation matrix illustrates the degree of connotation that exists between the physiological characteristics of plants, their water relationships, and the biochemical contents of wheat that has been subjected to salinity stress by seawater (SW) and treated with biofertilizers (BF). The upper matrix shows the Pearson coefficients, and the results were significant at ***p< 0.01, **p< 0.05, or *p< 0.1 as marked. The red solid lines in the lower matrix show a smooth regression between the two factors. CAT, Catalase; POD, Peroxidase; SOD, Superoxide dismutase; Chl, Total chlorophyll contents; CMS, Cell membrane stability; H2O2, Hydrogen peroxide; Pro, Proline; GB, Glycine betaine; MDA, Malondialdehyde; RWC, Relative water content.

Statistically significant associations were recorded between physiological characteristics, plant water status, and biochemical parameters through correlation analysis. Nevertheless, the strength and direction of these associations were contingent on the nature and severity of the treatments that were administered. As illustrated in **Figure 6**, chlorophyll content (Chl) exhibited strong positive associations with cell membrane stability (CMS) and relative water content (RWC), while demonstrating negative associations with glycine betaine (GB), proline, malondialdehyde (MDA), and reactive oxygen species (ROS). In consistent with Chl, the CMS varied in the opposite direction with ROS, osmolytic content, and plant water relations. On the other hand, the SOD activity depicted a positive association with GB, proline (**Figure 6**). Furthermore, the catalytic activity of CAT varied positively with proline. In general, the ROS, activity of antioxidant enzymes (CAT, POD, and SOD), and osmolytes (GB, proline, MDA) depicted significant positive association among them as shown in **Figure 6**. Both chlorophyll content (Chl) and relative water content (RWC) are decreased by the things listed above. A study of the effects of salinity and biofertilizer (BF) treatments on individuals showed that these treatments had different effects on trait interactions (**Figure 6**). Different salt treatments changed the connections between traits in different ways. For example, salinity levels significantly influenced the interrelationships among traits. Similarly, BF treatments altered these associations, indicating that the nature and strength of trait correlations depend on BF concentration.

**Figure 7:**
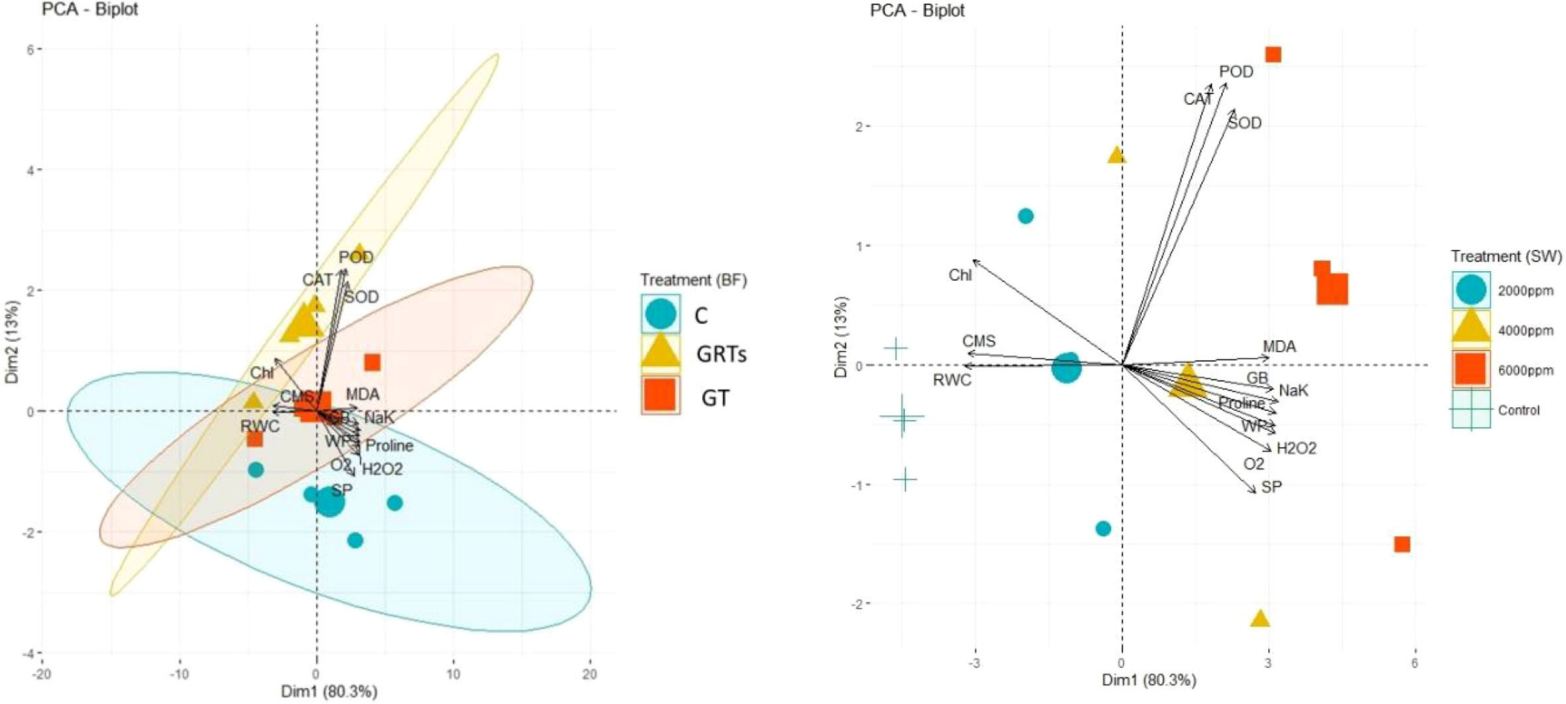
Principal component analysis (PCA) vectors illustrating the impact on the proximity correlation of biochemical constituents, physiological characteristics, and salinity stress treatments administered with saltwater. (SW) 0, 2000, 4000, 6000 ppm (right) and treatments of biofertilizers. C, Control; GT, Grain treatment; GRTs, Grain and root treatments (left).

The principal component analysis (PCA) results added to the correlation analysis results by showing how different salinity levels and BF treatments affect the levels and relationships of important physiological and biochemical factors, such as antioxidant enzymes, chlorophyll (Chl), chlorophyll a/b ratio (CMS), reactive oxygen species (ROS), osmolytes, and plant water status. As the salinity levels changed (SW0 = 0 ppm, SW1 = 2000 ppm, SW2 = 4000 ppm, and SW3 = 6000 ppm), the PCA biplot showed that the amounts and patterns of trait expression and association changed in different ways (**Figure 7**). The different lengths of the vectors that came from the biplot showed how sensitive different traits were to salt. The strength of the links between the trait vectors was shown by their distance apart. The closer the vectors were, the stronger the links were.

**Figure 8:**
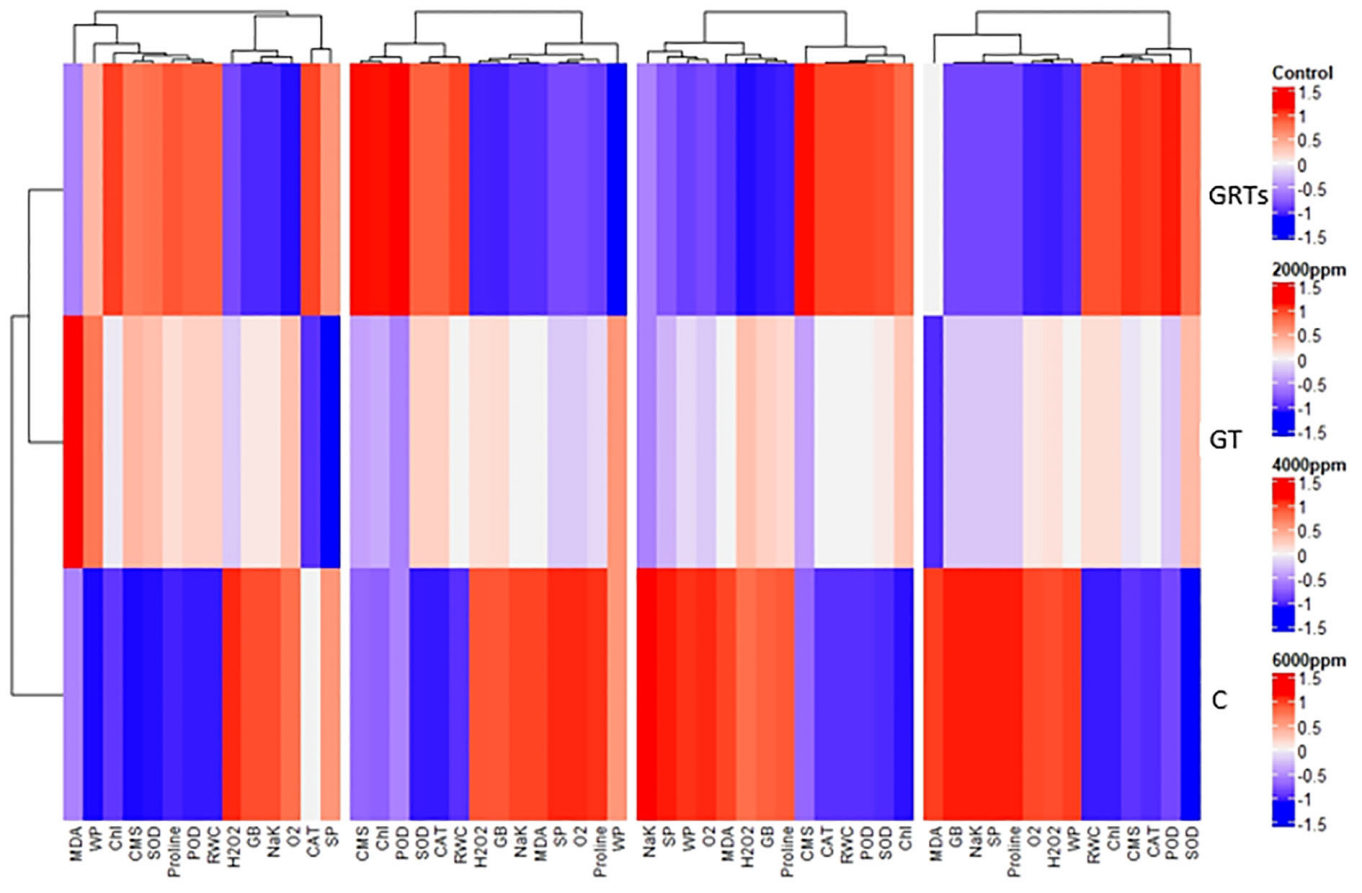
Cluster dendrogram heatmap depicting physiological and biochemical responses of wheat cultivar (*Yecora rojo*) to biofertilizer treatments under different salinity stress levels.

The results of PCA have been further confirmed by heatmap cluster analysis. In **Figure 8**, the different band patterns and cluster distribution show how the levels of biofilm (BF) change when the salinity stress levels (SW0 = 0 ppm, SW1 = 2000 ppm, SW2 = 4000 ppm, and SW3 = 6000 ppm) are changed. These levels affect the expression and association of chlorophyll (Chl), chloroplast movement (CMS), plant water relations, osmolytes, antioxidant enzymes, and reactive oxygen species (ROS). This is true for the same levels (C: Control, GT: Grain Treatment, GRTs: Grain and Root Treatment) of BF. This shows that different relationships between salinity and BF (salinity x BF) change the way characteristics are correlated and expressed. This was the next step to fix the PCA data. This was also shown by the combined heatmap study, which showed that each interaction between salinity and BF changes how attributes are expressed and linked in different ways. The heatmap analysis confirmed these findings by grouping features based on their link to changes in salt and how plants responded to biofertilizer treatments (**Figure 8**).

## 4 Discussion

Salinity stress is a significant constraint to wheat (*Triticum aestivum* L.) production, especially for spring wheat cultivated in semi-arid and arid areas, by causing disruption of physiological processes, oxidative damage, and repression of gene expression. In the current study, biofertilizers containing salutiferous rhizobacteria—*Azotobacter chroococcum*, *Bacillus megaterium*, and *Bacillus circulans*—that were applied as seed treatment and seed with root treatment were evaluated for their effectiveness in inducing salinity tolerance in spring wheat.

Chlorophyll content, relative water content (RWC), and cell membrane stability (CMS) significantly decreased under salinity stress, indicating impaired photosynthetic capacity and membrane integrity (Halder et al., 2022; Kumar et al., 2022). The biofertilizer treatments, nonetheless, effectively mitigated these effects, restoring these parameters to near-control levels (Ilyas and Naz, 2024). This improvement reflects more efficient osmotic adjustment and photosynthesis in inoculated plants, as reported in similar studies on wheat and barley or other crops (Bhardwaj et al., 2014; Glick, 2020; Sharma and Joshi, 2025; Sumbul et al., 2020; Vafa et al., 2021). Besides, salinity-induced osmolyte accumulation of proline and glycine betaine, necessary for osmoprotection and ROS scavenging, was controlled in the plants treated with biofertilizer (Gul et al., 2023). These treatments kept the osmolyte profile in check, reducing the metabolic cost of stress adaptation (Bashan et al., 2014; Egamberdieva, 2009; El Semary et al., 2020; Mahanty et al., 2017).

The use of biofertilizers significantly increased the antioxidant defense mechanisms (Upadhyay et al., 2019). While salinity alone caused increased activities of superoxide dismutase (SOD), peroxidase (POD), and catalase (CAT), enzyme activities were significantly higher in biofertilizer-treated groups, particularly in the combined group treatments (GRTs). This finding indicates a more efficient detoxification system for reactive oxygen species ROS (Borriss, 2011; Chen et al., 2022; Sabkia et al., 2021; Sharma et al., 2020). Concurrently, salinity stress increased the content of hydrogen peroxide (H_2_O_2_) and malondialdehyde (MDA), indicators of oxidative stress and lipid peroxidation. Biofertilizers significantly reduced these contents (Zhao et al., 2024), particularly under combined treatments, illustrating their protective function in guarding membrane stability and diminishing oxidative damage (Bashan and de-Bashan, 2010).

At the molecular level, the enhanced activation genes to tolerate stress —*TaSOD2, TaPOD-D1, TaCAT1, TaHKT1;4, TaNHX2, TaP5CS*, and *TaFER-5B*—was more pronounced in biofertilizer-treated plants (Kumar et al., 2020). This suggests enhanced activation of ion transport, antioxidant pathways, osmolyte biosynthesis, and ion homeostasis under salinity stress, similar to findings reported by (Bharti et al., 2016; Du et al., 2023; Glick, 2012; Ibáñez et al., 2023; Mahato and Kafle, 2018; Rocha et al., 2019). The concerted expression of genes by the biofertilizer indicates their capability to condition the wheat plants towards a superior, more efficient, and coordinated stress response.

The correlation matrix that we analyzed indicated high positive correlations between antioxidant enzymes (SOD, POD, CAT) and other physiological markers (chlorophyll content, RWC, CMS) and negative correlations with oxidative stress markers (hydrogen peroxide and MDA). Such correlations are supported by earlier studies (Bashan and de-Bashan, 2010; Maheshwari et al., 2013), which indicate the interconnectedness of enzymatic antioxidant defense mechanisms and stress mitigation in biofertilizer-treated plants. The PCA treatments were separated distinctly, with GRT and GT appearing as distinct clusters away from the untreated control under salinity stress. Loading plots indicated that the primary drivers of variation were photosynthetic pigments, water content, and antioxidant enzymes. This corroborates results from studies such as Bhattacharyya and Jha (2012) (Ibáñez et al., 2023), who noted the same trends in wheat and other crops inoculated with PGPR under stress. The heatmap visually confirmed these results through the clustering of positive characteristics (high antioxidant activity, low ROS production, and better physiological performance) against the GRT and GT treatments. This overall response confirms the systemic impact of biofertilizers and is in consensus with contemporary research that depicts the biofertilizer regulation of multiple stress-response pathways (Kumar et al., 2022; Mahanty et al., 2017; Sabkia et al., 2021).

The mechanistic explanation for the physiological enhancements observed in wheat plants treated with biofertilizer is the increased expression of critical stress-responsive genes. For example, the upregulation of TaHKT1;4 and TaNHX2, which are responsible for maintaining ion homeostasis under salinity stress, is consistent with the restoration of chlorophyll content, relative water content, and membrane stability (Ayaz et al., 2022; Abu-Qaoud et al., 2021). In the same vein, the increased expression of antioxidant genes, including TaSOD2, TaPOD-D1, and TaCAT1, is directly correlated with the reduction in oxidative damage (low H_2_O_2_ and MDA), resulting in more efficient detoxification of ROS and increased enzymatic activity (Upadhyay et al., 2019; Bashan and de-Bashan, 2010). The activation of TaP5CS and TaFER-5B, which regulate proline biosynthesis and redox stability, is associated with the balanced osmolyte accumulation observed in inoculated plants (Ábrahám et al., 2010; Valadez-Bustos et al., 2016). Therefore, the coordinated gene expression profiles establish a molecular foundation for the physiological resilience that PGPR induces, indicating that the enhanced antioxidant capacity, osmotic adjustment, and ion regulation at the physiological level are supported by targeted transcriptional responses (Bharti et al., 2016; Kumar et al., 2020; Al Khallaf et al., 2024).

## 5 Conclusion

In conclusion, current research established that co-inoculation with *Azotobacter chroococcum, Bacillus megaterium*, and *Bacillus circulans* markedly improves the salt tolerance of spring wheat *(Triticum aestivum L.* cv. *Yecora Rojo*). The use of biofertilizer reinstated essential physiological characteristics, such as chlorophyll concentration, membrane integrity, and hydration levels, while mitigating oxidative damage through enhanced antioxidant function and regulated osmolyte levels. The activation of stress-responsive genes related to ion homeostasis, osmotic adjustment, and reactive oxygen species detoxification at the molecular level further substantiated the protective function of microbial inoculation. The combined application of grain and root (GRT) provided the most significant effects, highlighting the synergistic potential of dual inoculation. The findings underscore the potential of microbial consortia as a sustainable and ecologically viable method for mitigating salinity stress, enhancing crop resilience, and increasing production in salt-affected soils.

## Data Availability

The datasets presented in this study can be found in online repositories. The names of the repository/repositories and accession number(s) can be found in the article/supplementary material.
